# P03-026 – Sweet’s syndrome: report of a new case

**DOI:** 10.1186/1546-0096-11-S1-A224

**Published:** 2013-11-08

**Authors:** M Mael-Ainin, H Boudhir, A Saidi, K Senouci, B Hassam

**Affiliations:** 1 Dermatologie - Vénérologie, Centre Hospitalier Universitaire IBN Sina, Morocco; 2 Centre of Anatomical Pathology, United Nations, Rabat, Morocco

## Introduction

Sweet syndrome is a neutrophilic dermatosis usually idiopathic; it may be associated in one third of cases with inflammatory or neoplastic diseases, or subsequently to drug intake. Its association with pregnancy has been rarely described. Through this observation, we report the case of a pregnancy discovered by the etiological diagnosis of Sweet's syndrome.

## Objectives

To emphasize on the importance of seeking pregnancy during etiological Sweet's syndrome in a young woman of childbearing age.

## Methods

Mrs. S.H 31 years old, whose antecedents show irregular menstrual cycles, mechanical contraception used in anarchic way, was hospitalized for painful nodular erythematous papulopustular lesions located in the face and limbs, associated with fever (38.5°C) and arthralgia of the ankles. Clinical exam showed limited papulo-nodular erythematous lesions of variable size, in the face, the upper limbs and thighs with the dermal-hypodermic nodule in legs.

Tests showed neutrophilic leukocytosis at 13000 elements/mm3, a sedimentation rate accelerated to 100mm in the first hour and a C-reactive protein increased to 79mg/l. Liver function was normal and 24h proteinuria was negative.

Skin biopsy was in favor of Sweet's syndrome. An etiological test was conducted. No abnormality was observed for blood smear, electrophoresis of proteins, lactate dehydrogenase dosage and carcinoembryonic antigen. Given the gynecological history of the patient, a dosage of Beta-HCG was performed and showed an increase to 119358mUI/ml. Endovaginal ultrasonography showed mono fetal pregnancy with a gestational age estimated to six weeks. The diagnosis of pregnancy Sweet Sydrome was retained. Therapeutic abstention was advocated with clinical monitoring and the evolution was spontaneously favorable after 6 weeks.

## Results

Sweet's syndrome in pregnancy represents only 2% of all etiologies of this neutrophilic dermatosis. Its pathogenesis, likely due to a hormonal mechanism, remains unclear. Fetal-maternal prognosis is not affected by the occurrence of Sweet's syndrome, the only risk is the recurrence in subsequent pregnancies. Treatment consists mainly on corticosteroids at a dose of 0.5 to 1mg/kg/jour allowing apyrexia and rapid regression of symptoms. In our case, no therapy has been advocated giving the conservation of the general condition of the patient, which resulted in a good evolution.

## Conclusion

This observation shows that Sweet's syndrome pregnancy may occur at a very early gestational age. Seeking pregnancy is thus necessary in any patient of childbearing age with Sweet's syndrome to avoid exhaustive etiological and allow appropriate treatment.

## Disclosure of interest

None declared

